# Polygonal Area of Retinal Pigment Epithelium Avulsion Following Retinal Cryotherapy for Retinal Detachment

**DOI:** 10.1155/crop/7456188

**Published:** 2026-03-20

**Authors:** Georgios Mylonas, Gregor S. Reiter, Markus Ritter, Gabor G. Deak, Andreas Pollreisz, Stefan Sacu, Michael Georgopoulos

**Affiliations:** ^1^ Department of Ophthalmology, Medical University of Vienna, Vienna, Austria, meduniwien.ac.at

## Abstract

**Purpose:**

This study is aimed at reporting a rare case of polygonal retinal pigment epithelium (RPE) avulsion following retinal cryotherapy for retinal detachment.

**Methods:**

Case report.

**Results:**

A 53‐year‐old man presented with recurrent retinal detachment 30 days after 23G vitrectomy with 20% sulfur hexafluoride (SF6) gas endotamponade. Intraocular pressure was < 1 mmHg, with horizontal chorioretinal folds due to hypotony. Re‐vitrectomy with PFCL, cryotherapy, endolaser, and silicone oil was performed. During surgery, a large RPE avulsion with island formation and a small full‐thickness retinal avulsion causing re‐detachment were identified in the cryotherapy area. After 6 months with attached retina, the silicone oil was removed and the retina remained stable.

**Conclusions:**

Cryotherapy‐induced scarring for retinal detachment repair is likely to cause traction. In our case, traction from the scar resulted in a full‐thickness retinal tear and RPE avulsion, leading to re‐detachment. Careful follow‐up is essential to detect and treat complications.

## 1. Introduction

Retinal cryotherapy is the most commonly used method for creating chorioretinal adhesion after laser photocoagulation in conventional retinal reattachment surgery [1, 2]. Cryotherapy is easy to use, allows rapid application, and can be delivered through subretinal fluid, which makes it the preferred method for some surgeons. This procedure aims to create a chorioretinal scar to seal retinal breaks, treat retinal tumors, or reduce retinal neovascularization. While the exact mechanism of injury from intracellular freezing is not fully understood, it is believed to involve physical damage to organelle and plasma membranes [3]. The formation of a chorioretinal scar to close retinal breaks is a critical component of retinal reattachment surgery. The strength of the adhesion between the retina and the retinal pigment epithelium (RPE) is generally proportional to the intensity of the cryotherapy application [4, 5]. Cryotherapy has been shown to disrupt the blood–ocular barrier, allowing pigment epithelial cells to disperse into the subretinal space, pass through retinal breaks, and enter in to the vitreous cavity—a process thought to play a key role in the development of proliferative vitreoretinopathy (PVR) [6, 7]. Several complications have been reported with retinal cryotherapy, including vitreous hemorrhage, transient rise in intraocular pressure, macular edema, excessive chorioretinal scarring, PVR, formation of new retinal breaks, choroidal detachment, and cataract formation, with retinal necrosis occurring rarely. Very rare but serious complications include scleral perforation and inadvertent posterior pole cryo‐application [8–11].

## 2. Case presentation

A 53‐year‐old pseudophakic Caucasian male presented to our department with a scotoma in his visual field and the sudden appearance of many floaters. The patient had undergone vitrectomy with gas endotamponade and cryotherapy for retinal detachment repair in the same eye 40 days previously. During the procedure, cryotherapy was applied until complete retinal freezing was achieved. Three cryotherapy applications were delivered to the main retinal avulsion at the 9 o’clock position, with additional single cryotherapy applications applied to two small retinal tears at the 7 and 8 o’clock positions. He had also previously undergone uncomplicated cataract extraction. Examination revealed a corrected distance visual acuity (CDVA) of 0.25. Slit‐lamp examination revealed a normal anterior segment, and intraocular pressure in the right eye measured by applanation tonometry was < 1 mmHg. Dilated funduscopic examination revealed a recurrent retinal detachment in the area of the old retinal tear and cryotherapy scars (Figure 1a). Horizontal chorioretinal folds were also present (Figure 1a). Interestingly, the day before, the patient had presented to our department for a scheduled follow‐up visit after the initial retinal detachment surgery, where the CDVA was 0.8, the intraocular pressure was normal and the dilated funduscopic examination revealed an attached retina.

Figure 1(a) Ultrawide field fundus (UWF) image of the right eye 1 month after the initial surgery, showing recurrent retinal detachment in the temporal area. Note the edge of the retinal pigment epithelium avulsion (black arrows) and the chorioretinal folds due to hypotony (red arrows). (b) UWF image at 3 months after silicone oil removal showing a retinal pigment epithelium avulsion with “island” formation. The retina remained attached.

**Figure:**
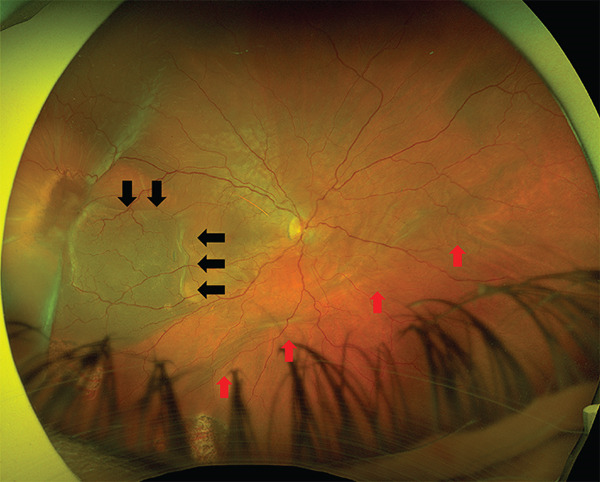
(a)

**Figure:**
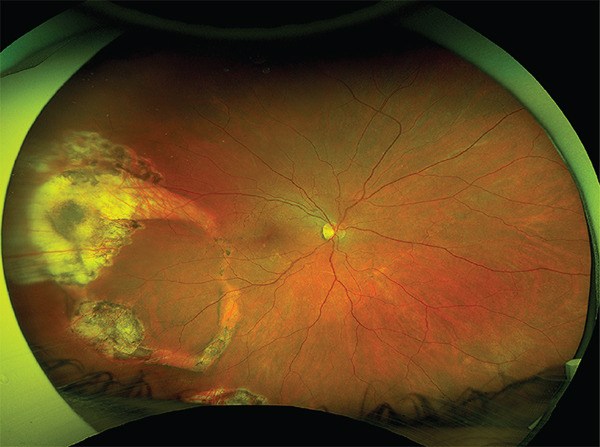
(b)

The patient was scheduled for further surgery to repair the recurrent retinal detachment. We performed a re‐vitrectomy with perfluorocarbon liquid (PFCL), cryotherapy, endolaser, and silicone oil (SO) was used as a long‐term endotamponade. During retinal flattening with PFCL, we observed a closed RPE avulsion and the formation of an RPE “island” that remained attached to the neurosensory retina while floating on the subretinal fluid, resulting from RPE fracture (Figures 1 and 2). The retina was attached to the RPE in this area, but the RPE was completely detached from the underlying choroid. There was also a small tear in the area of the old cryotherapy scar. Endolaser and cryotherapy were used to create chorioretinal adhesion and SO was used as a long‐term endotamponade. Retrospectively, we studied the preoperative OCT images and noticed the area of RPE avulsion, characterized by the elevation of both the RPE and retinal layers (Figure 2a).

Figure 2(a and b) Preoperative infrared and OCT images, respectively, showing retinal pigment epithelium detachment (red arrows) and neurosensory retinal detachment (green arrows). (c and d) Infrared and OCT images, respectively, 1 month after surgery under silicone oil. Note the area of retinal pigment epithelium avulsion and the corresponding absence of the retinal pigment epithelium in the OCT scan (yellow arrows).

**Figure:**
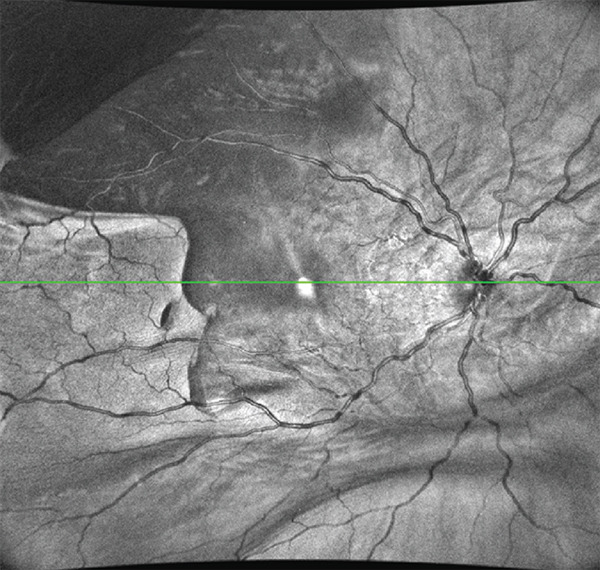
(a)

**Figure:**
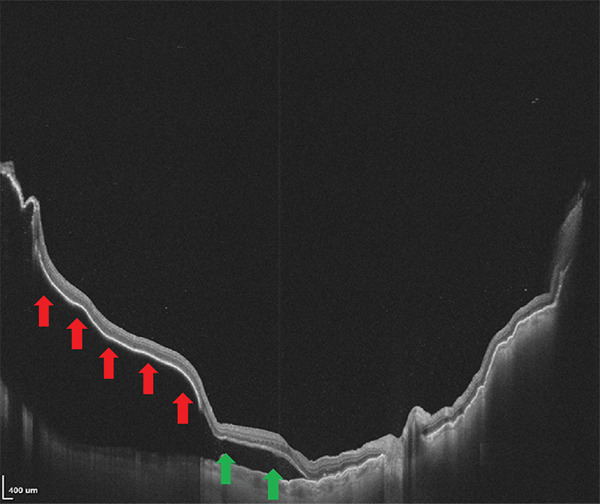
(b)

**Figure:**
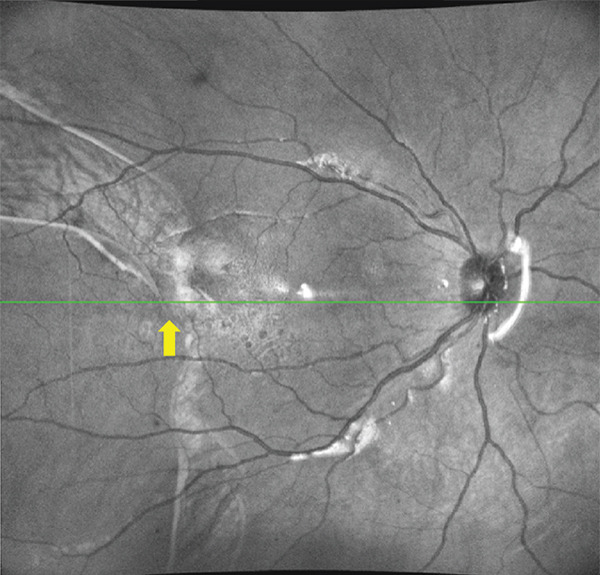
(c)

**Figure:**
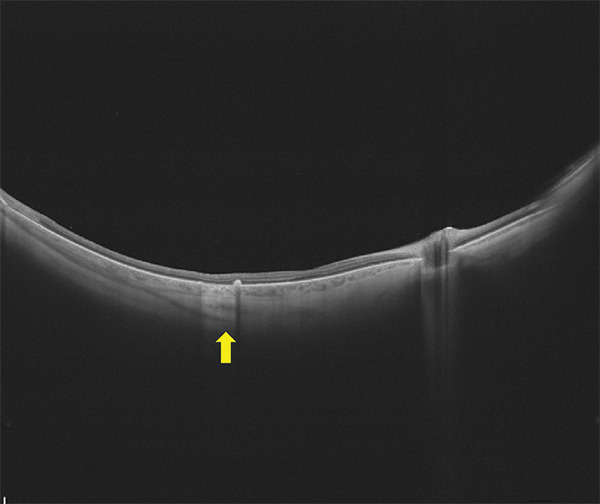
(d)

At the 6‐month follow‐up, the retina remained fully attached with no evidence of PVR, and the decision was made to proceed with the removal of the SO. At the 3‐month follow‐up post‐removal, the retina remained attached, and visual acuity was recorded at 0.4 (Figure 1b und 2b).

## 3. Discussion

Although retinal cryotherapy for the treatment of retinal tears is generally safe, several complications have been reported, including intravitreal dispersion of viable retinal pigment epithelial cells and extension of retinal tears through the cryosurgical scar [8–10]. In addition, the formation of new retinal tears in cryosurgical scar tissue has been documented as a late complication of cryotherapy for retinopathy of prematurity [8, 9].

When cryotherapy was applied to the pigment epithelium and retina, strong adhesion occurred, characterized by the formation of true cell junctions between the pigment epithelium and retinal cells. In our case, it appears that the contraction of the cryosurgical scar caused a closed area of RPE avulsion, leading to detachment of both RPE and retina. The exposure of large choroidal vessels with significant pumping capacity to the subretinal fluid may explain the extensive hypotony and horizontal chorioretinal folds. Furthermore, the contraction of the retinal tissue after cryotherapy could cause the new retinal tear and recurrence of the retinal detachment.

In the second surgery, we attempted to repair the retina using both cryotherapy and laser photocoagulation (LK). LK relies on an intact RPE, as melanin in the RPE absorbs laser energy and facilitates adhesion between the retina and the underlying choroid. In contrast, cryotherapy is less dependent on an intact RPE, although its healing process still benefits from the RPE response. While the RPE contributes to overall healing, cryotherapy induces adhesion primarily through direct tissue freezing and inflammation‐driven scarring rather than RPE‐mediated laser absorption. Both methods are effective when applied to the pigment epithelium and retina. However, in our case, an RPE tear resulted in the absence of RPE around the retinal hole. Given this limitation, we chose to use SO as a long‐term endotamponade to stabilize the retina and promote adhesion in the absence of an intact RPE.

Retinal cryotherapy is indeed effective for the treatment of various retinal diseases. Although it is generally safe when used judiciously, there are potential complications such as the formation of new retinal holes and recurrence of retinal detachment, hemorrhage and inflammation, especially when used extensively over the entire retina. Because of these risks, careful follow‐up of patients undergoing retinal cryotherapy is essential to detect complications early and effectively manage any adverse effects.

## Funding

No funding was received for this manuscript. Open Access funding provided by Medizinische Universitat Wien/KEMÖ.

## Conflicts of Interest

The authors declare no conflicts of interest.

## Data Availability

The data that support the findings of this study are available from the corresponding author upon reasonable request.
